# The Value of Brain Imaging and Electrophysiological Testing for Early Screening of Autism Spectrum Disorder: A Systematic Review

**DOI:** 10.3389/fnins.2021.812946

**Published:** 2022-02-03

**Authors:** Cullen Clairmont, Jiuju Wang, Samia Tariq, Hannah Tayla Sherman, Mingxuan Zhao, Xue-Jun Kong

**Affiliations:** ^1^Synapse Lab, Athinoula A. Martinos Center, Massachusetts General Hospital, Boston, MA, United States; ^2^NHC Key Laboratory of Mental Health, National Clinical Research Center for Mental Disorders, Peking University Sixth Hospital, Beijing, China; ^3^Department of Business Analytics, Bentley University, Waltham, MA, United States; ^4^Department of Psychiatry, Beth Israel Deaconess Medical Center, Boston, MA, United States

**Keywords:** autism spectrum disorder (ASD), early screening, magnetic resonance imaging (MRI), electroencephalogram (EEG), functional near-infrared spectroscopy (fNIRS)

## Abstract

Given the significance of validating reliable tests for the early detection of autism spectrum disorder (ASD), this systematic review aims to summarize available evidence of neuroimaging and neurophysiological changes in high-risk infants to improve ASD early diagnosis. We included peer-reviewed, primary research in English published before May 21, 2021, involving the use of magnetic resonance imaging (MRI), electroencephalogram (EEG), or functional near-infrared spectroscopy (fNIRS) in children with high risk for ASD under 24 months of age. The main exclusion criteria includes diagnosis of a genetic disorder and gestation age of less the 36 weeks. Online research was performed on PubMed, Web of Science, PsycINFO, and CINAHL. Article selection was conducted by two reviewers to minimize bias. This research was funded by Massachusetts General Hospital Sundry funding. IRB approval was not submitted as it was deemed unnecessary. We included 75 primary research articles. Studies showed that high-risk infants had divergent developmental trajectories for fractional anisotropy and regional brain volumes, increased CSF volume, and global connectivity abnormalities on MRI, decreased sensitivity for familiar faces, atypical lateralization during facial and auditory processing, and different spectral powers across multiple band frequencies on EEG, and distinct developmental trajectories in functional connectivity and regional oxyhemoglobin concentrations in fNIRS. These findings in infants were found to be correlated with the core ASD symptoms and diagnosis at toddler age. Despite the lack of quantitative analysis of the research database, neuroimaging and electrophysiological biomarkers have promising value for the screening of ASD as early as infancy with high accuracy, which warrants further investigation.

## Introduction

Autism spectrum disorder (ASD) is a developmental disorder characterized by deficits in social interaction and repetitive and restrictive behaviors. The prevalence of ASD diagnosis has been steadily rising for decades; as of the most recent report in 2016, 1 in 54 children will be diagnosed with ASD by the age of 8 (Maenner, 2020), and, between 2002 and 2004, the average age of ASD diagnosis in Medicaid-enrolled children was not until 64.9 months (Mandell et al., 2010). Clinical diagnosis of autism is typically not reliable until after 24 months of age (Sanchack and Thomas, 2016). Current clinical diagnosis of ASD is based on the presentation of core symptoms (Sanchack and Thomas, 2016). The Autism Diagnostic Observation Schedule, Second Edition (ADOS-2), commonly referred to as the gold standard diagnostic tool for autism, assesses the clinical presentation of these symptoms, in order to guide ASD diagnosis (Carr, 2013).

Various psychometric screening instruments have been proposed and applied to improve early detection and diagnosis in ASD (Levy et al., 2020; Petrocchi et al., 2020). In recent years, information and communication technologies (ICT) related products such as smartphones, tablets, eye trackers, or robots (Desideri et al., 2021), home video (Tariq et al., 2018), multi-modular AI approaches (Abbas et al., 2020), and machine learning strategies (Achenie et al., 2019) have offered easier and comparable alternative psychometric screening for ASD. However, these psychometric screening methods are not readily implemented during infancy.

In recent years, early biological indicators of ASD have been studied even in infancy (Frye et al., 2019). In addition to genetic, immunological, and metabolic biomarkers, brain imaging and neurophysiology biomarkers may have diagnostic utility during infancy. Because ASD diagnosis is not yet possible in infants, most research into the early signs of ASD divides research populations into infants with high familial risk (HR infants) and infants without familial risk (LR infants). Recent research Hazlett et al. (2017) tested 106 HR infants and 42 LR infants for ASD in a longitudinal study using a deep learning algorithm to measure the surface area on Magnetic Resonance Imaging (MRI); this study was conducted in a cohort of ASD individuals at 6–12 months to predict the diagnosis at 24 months and found to have 88% sensitivity and 81% positive predictive value (Hazlett et al., 2017). Emerson et al. (2017) tested 59 infants with high risk for ASD in a longitudinal study and found functional connectivity at 6 months with a machine-learning algorithm predicted ASD diagnosis at 24 months with 81.8% sensitivity (Emerson et al., 2017). Other longitudinal studies have leveraged electroencephalogram (EEG) (Kolesnik et al., 2019) and functional Near-Infrared Spectroscopy (fNIRS) (Kolesnik et al., 2019; Zhang and Roeyers, 2019) during infancy to predict ASD diagnosis later in life. Neurological differences between HR infants who are later diagnosed with ASD (HR-ASD) and HR infants who test negative for ASD (HR-TD) must be studied, so that early detection becomes more accurate and can be clinically applied.

Detection of abnormal brain development and neurological activity in HR-ASD infants with neuroimaging and neurophysiological methods offers a promising avenue for early screening by directly reflecting the early pathological changes related to later development of ASD. The new findings in this field have not yet been reported or emphasized. We will focus on MRI, EEG, and fNIRS, the three most used and reported neuroimaging and neurophysiological tools in the field for early ASD screening. This systematic review aims to summarize available evidence from primary research of these three neuroimaging and neurophysiological methods to characterize neurological differences in HR infants and compare the pros and cons of each modality in ASD early screening.

## Materials and Methods

### Inclusion and Exclusion Criteria

The study was conducted in the absence of an Internal Review Board approved protocol. The inclusion criteria was established and applied as follows: all articles were published in English; all articles were peer-reviewed, primary research; all articles conducted empirical research that involved the use of EEG, MRI, or fNIRS in children with HR for ASD; all articles had a scanning age of 24 months of age or younger; all articles grouped infants based on risk for developing ASD. The exclusion criteria was established and applied as follows: No articles included participants with genetic disorders, including but not limited to epilepsy, Fragile X syndrome, Rett syndrome, tuberous sclerosis, and cerebral palsy; no articles included infants with a gestational age of less than 36 weeks; no articles were designed not to report findings, such as methodological articles.

### Literature Research Strategy

We conducted a comprehensive search for studies that focused on the developmental stage of infants and toddlers under age of 2 years and at risk for ASD. We focused on studies that used EEG, MRI or fNIRS. All articles were published before May 21st, 2021. The online research was performed on PubMed, Web of Science, PsycINFO, and CINAHL. Using keyword searches across four scientific databases, a total of 943 articles were initially considered for this systematic review. All articles evaluated in this study were gathered through searching for three keyword domains. First, the article needed to contain either “autism” or “ASD.” Second, the article needed to contain a word or phrase that indicated that participants had a high risk of developing ASD, such as “at risk” or “familial risk.” Third, the article needed to contain a keyword related to either MRI, EEG, or fNIRS.

**FIGURE 1:**
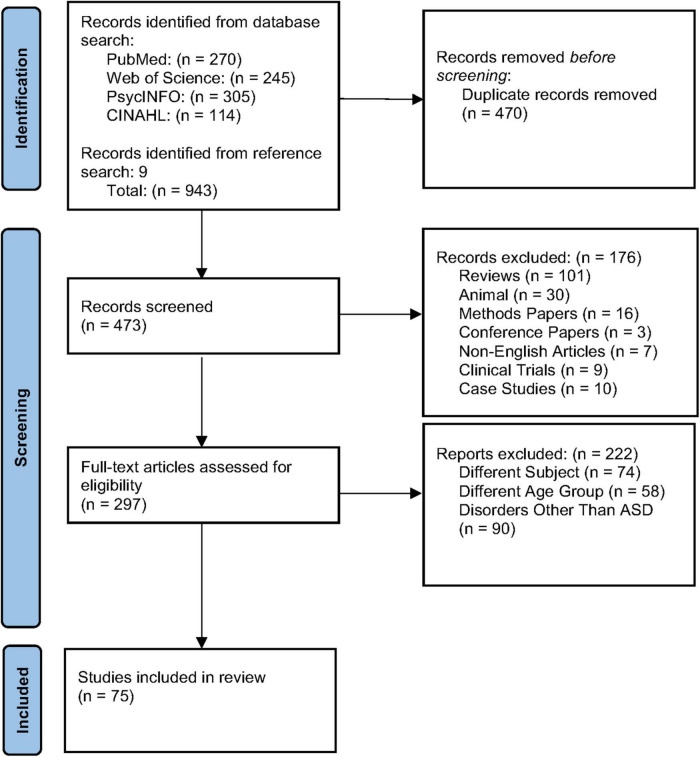
PRISMA diagram of literature screening and selection.

The following types of articles were excluded during initial screening: anecdotal case reports of a single or few patients, analysis of clinical database or registries, review articles, commentaries, mechanism of action research articles, meta-analysis or systematic reviews, conference papers, preclinical and animal studies, interventional clinical trials, and studies designed not to find meaningful results. Titles and abstracts of the papers identified by this initial search strategy were evaluated by two independent reviewers. We obtained potentially relevant papers and conducted subsequent full text screens. From the database search, 66 articles were selected to be reviewed. We also conducted a recursive search of the literature based on the bibliographies of the relevant full text articles. Nine additional articles were identified, screened, and included based on that search of references in relevant full text articles. Two independent reviewers conducted full text screens using pre-designed eligibility criteria. Any disagreement between investigators was resolved by consensus. One paper, Stahl et al. (2012), was categorized as a methodological paper because it used the same dataset as Elsabbagh et al. (2009) and reported similar findings, so its contribution to the field was determined to be methodological in nature and was excluded (Elsabbagh et al., 2009; Stahl et al., 2012). Two papers, Brito et al. (2019) and Riva et al. (2019), while very similar to included articles, did not group infants by ASD risk at the start of the experiment and were categorized as the wrong subject matter and excluded (Brito et al., 2019; Riva et al., 2019). See Figure 1 for the details.

### Assessment of Risk of Bias

Steps were taken by reviewers to minimize bias in this systematic review. Search terms were created prior to the start of the systematic review and were executed across four scientific databases. Search terms were optimized through the consensus of all reviewers. Inclusion and exclusion criteria is clearly stated and was appropriately applied to all articles. The quality or interpretation of article findings were not evaluated during the exclusion process. At least two reviewers achieved consensus on all included articles. The literature research strategy was followed closely, and no protocol deviations occurred to the knowledge of the reviewers. Because of the diversity of research included in this systematic review, no quantitative meta-analysis was conducted.

### Data Extraction

Material information has been extracted from each and every selected study. One reviewer extracted individual imaging or electrophysiology data from each study (sample size, mean, standard deviation, and percentages of samples of ASD and control groups) into a pre-piloted Microsoft Excel spreadsheet (XP professional edition; Microsoft Corp., Redmond, WA, United States). A second reviewer cross-checked each data variable extracted against the original publication. Disagreement was resolved via consensus. In addition, the following information was extracted wherever available: age of participants, gender of participants, imaging or electrophysiology methodology, and definition of positive result. When necessary, we contacted the authors to obtain original data via e-mail.

## Results

**FIGURE 2:**
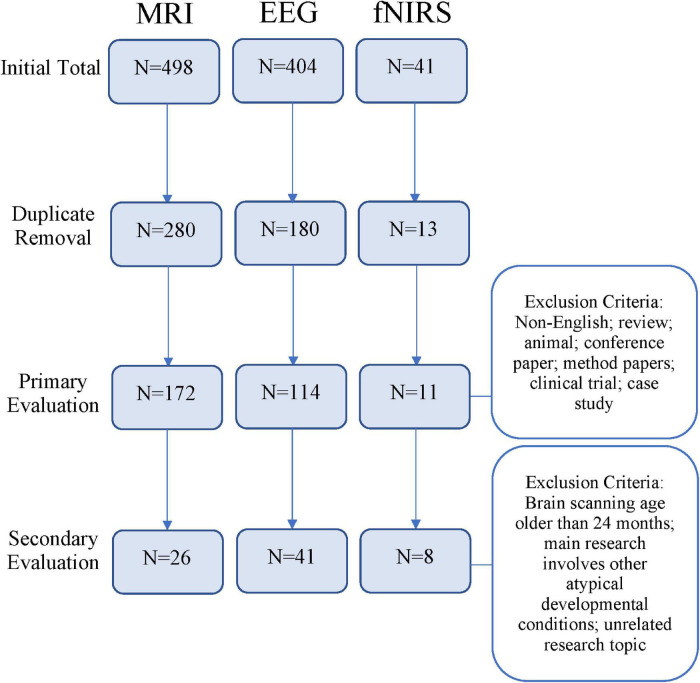
Flowchart depicting article screening process based on research methods [magnetic resonance imaging (MRI), electroencephalogram (EEG), or functional near-infrared spectroscopy (fNIRS)].

A total of 473 unique studies were identified, after excluding duplicates. Following a primary evaluation of titles and abstracts and exclusion based on article type, 297 articles were further assessed for eligibility. In the secondary evaluation, an additional 222 articles were excluded because of the exclusion criteria enumerated in Figure 1. Finally, 75 articles met our inclusion criteria for the present systematic review, including 26 articles using MRI, 41 EEG, and 8 fNIRS. See Figure 2 for further detail about MRI, EEG, and fNIRS article screening.

### Magnetic Resonance Imaging Studies

Magnetic resonance imaging (MRI) is a non-invasive and sophisticated imaging technique that uses strong magnetic fields to generate two dimensional image slices of the human body. The MRI discussed in this review will be limited to imaging of the brain, although the application therein will be diverse. Namely, MRI can be carried out to assess structure, using methods such as diffusion-weighted imaging, or function, through methods such as BOLD imaging. Twenty-six MRI studies were selected and are reviewed below. Twenty structural studies and six functional studies have been reviewed.

#### Structural Studies

##### White Matter Tracts

Substantial investigation into white matter structures via analysis of fractional anisotropy (FA), a measurement of diffusion properties in tissues, has revealed divergent trajectories between HR infants who later develop ASD (HR-ASD) and HR infants who do not. HR-ASD infants were found to have greater FA values at 6 months, before exhibiting slower FA growth until 24 months for bilateral limbic (fornix) and association (inferior longitudinal fasciculus and uncinate fasciculus) fiber tracts (Wolff et al., 2012), and ASD toddlers continued to display slower age-related changes in FA after 24 months (Solso et al., 2016). Enriched FA in the right superior longitudinal fasciculus at 6 weeks of age in HR infants was associated with more severe ASD symptoms at 36 months (Liu et al., 2019). Starting at 6 months, HR-ASD infants displayed elevated corpus callosum area and thickness, particularly in the anterior corpus callosum (Wolff et al., 2015). Longitudinal analysis from 6 to 24 months revealed that cerebellar and callosal white matter tracts were associated with repetitive behaviors and sensory responsiveness but not social deficits (Wolff et al., 2017). While HR-ASD infants had delayed visual-orienting latencies, LR infants exhibited an association between microstructural organization in the corpus callosum and visual-orienting latencies (Elison et al., 2013).

Network efficiency, measured by the strength of association and length of white matter tracts between regions, has been found to be reduced in HR infants. Lewis et al. (2014) reported that global efficiencies in the temporal, parietal, and occipital lobes, and Broca’s area were reduced in HR-ASD infants (Lewis et al., 2014). Further, these global inefficiencies were negatively correlated to ASD symptom severity across all HR infants (Lewis et al., 2014). A follow-up study by the same research group found local network inefficiencies in the right auditory cortex at 6 months and global network inefficiencies across Broca’s area at 12 months, which were both associated with symptom severity at 24 months (Lewis et al., 2017). Notably, machine learning techniques that use FA measurements and network connectivity have been implemented to generate an ASD classifier with an accuracy of 76% (Jin et al., 2015).

##### Cerebrospinal Fluid (CSF) and Regional Volumes

Increased extra-axial CSF starting at 6 months (Shen et al., 2013) and persisting through 24 months (Shen et al., 2017) has been observed in HR-ASD. Predicting ASD diagnosis using 6 months extra-axial volume at 6 months had an accuracy of 69% (Shen et al., 2017). While generalized cerebral cortical overgrowth was observed in 2-year-old toddlers with ASD (Hazlett et al., 2011), a follow-up article by the same research group reported that HR infants at 6 months old presented with no significant differences in gross regional volumes (Hazlett et al., 2012). Hazlett et al. (2017) found that HR-ASD infants experienced cortical surface area hyper-expansion between 6 and 12 months, then brain volume overgrowth between 12 and 24 months, which was associated with social deficits at 24 months of age. These researchers leveraged surface area information from 6 and/or 12 months to predict the autism diagnosis at 24 months and achieved an accuracy of 81% (Hazlett et al., 2017). While Cárdenas-de-la-Parra et al. (2020) agrees that HR-ASD infants experience overall brain volume overgrowth after 12 months of age, they argue that, compared to LR and HR-TD infants, HR-ASD infants actually have increased growth in white matter areas, including the splenium of the corpus callosum, and decreased growth in gray matter areas, including the right fusiform gyrus and inferior temporal gyrus (Cárdenas-de-la-Parra et al., 2020).

More recent research builds upon the findings from Hazlett et al. (2017) and delineates significant relationships between abnormal brain volumes and ASD symptomology. Infants between 4 and 6 months old were found to have significantly larger cerebellar and subcortical volumes, which were associated with core repetitive behaviors at 36 months (Pote et al., 2019). Subcortical volumes at 12 months have been associated with language skills at 24 months (Swanson et al., 2017). The amygdala and hippocampus were found to be enlarged in HR infants and toddlers between the ages of 6 and 24 months (Li et al., 2019). HR-ASD infants were also found to not only have more difficulty sleeping, but these sleeping difficulties were found to be directly related to hippocampal volume (MacDuffie et al., 2020).

#### Functional Studies

Two studies established differences in functional connectivity development between HR and LR infants in 1.5-month-old infants. Liu et al. (2020a) reported decreased connectivity between the temporal and somatosensory regions at 1.5 months but hyperconnectivity between these regions and decreased interhemispheric connectivity at 9 months old (Liu et al., 2020a). Nair et al. (2021) found that HR infants at 1.5 months had under- and overconnectivity in different thalamic pathways and that these aberrant connectivities were correlated with atypical social development at prior to 36 months. Functional connectivity at 6 months of age correlated with psychometric scores related to social behavior, language, motor development, and repetitive, ritualistic, and stereotyped behavior at 24 months of age (Emerson et al., 2017; McKinnon et al., 2019). Functional connectivity data collected at 6 months successfully predicted ASD with a positive predictive value of 100% (Emerson et al., 2017).

Few studies have examined fMRI activity in response to stimuli. Blasi et al. (2015) found that, when presented with auditory stimuli during sleep, only LR infants demonstrated specialization for human voices in the temporal and medial regions and exhibited stronger sensitivity to sad vocalizations in the right fusiform gyrus and left hippocampus (Blasi et al., 2015). Notably, HR infants, unlike the LR infants, demonstrated a correlation between social engagement and degree of voice specialization, indicating the potential importance of specialization in HR infants. Similarly, Liu et al. (2020b) found greater activity in the left amygdala and learning-relating signaling in the left temporal region in the LR group, but this signaling in the left temporal region at 9 months was negatively correlated with ASD symptom severity at 36 months (Liu et al., 2020b).

In summary, while HR-ASD infants’ white matter tracts develop rapidly up until 6 months of age and then more slowly through toddlerhood compared to HR-TD and LR children, they simultaneously exhibit gray matter overgrowth after 6 months, both on the whole and in several regions in particular, such as the amygdala and hippocampus. HR-ASD infants have complex, abnormal connectivity, characterized by structural, global inefficiency and functional over- and underconnectivity along multiple major neural pathways. The different developmental trajectory of white matter, gray matter, and functional connectivity in HR-ASD infants have all been significantly associated with the core symptoms of ASD, including stereotyped behavior and social function. See Table 1 for details about each MRI article reviewed.

**TABLE 1:** Magnetic resonance imaging (MRI) studies on early screening of autism spectrum disorders.

References	Age	High-risk group		Low-risk control group		Region of interest/task	Main findings
					
		Total no. (M/F)	HR-ASD no.	No. (M/F)	LR-ASD no.		
Wolff et al., 2015	6, 12, and 24 months	270 (169/101)	57 (47/10)	108 (66/42)	–	White matter structure	(1) HR-ASD showed significantly increased corpus callosum area and thickness starting at 6 months of age, especially in the anterior corpus callosum at 6 and 12 months; (2) Measures of area and thickness in the first year of life were correlated with repetitive behaviors at age 2 years; (3) Area and thickness differences between groups diminish by age 2 years.
Wolff et al., 2012	6, 12, and 24 months	92 (59/33)	28 (21/7)	–	–	White matter structure	(1) Fractional anisotropy trajectories for 12 of 15 fiber tracts differed between HR-ASD and HR-no-ASD; (2) HR-ASDs was characterized by higher fractional anisotropy values at 6 months followed by slower change over time relative to HR-no-ASD; (3) Atypical white matter pathways may develop before the presentation of ASD symptoms in the first year of life.
Wolff et al., 2017	6, 12, and 24 months	217 (139/78)	44 (39/5)	–	–	White matter structure	(1) Repetitive behavior and sensory responsiveness strongly correlated in children with ASD; (2) Longitudinal analysis finds genu and cerebellar pathways are correlated with repetitive behavior and sensory responsiveness.
Solso et al., 2016	12–48 months	–	61 (48/13)	33 (20/13)	–	White matter structure	(1) Abnormalities of multiple frontal axon pathways at the age of first clinical signs of ASD; (2) White matter tracts in the frontal regions of ASD toddlers were greater in FA and volume at 1–2 years.
Jin et al., 2015	0–24 months	40 (29/11)	–	40 (27/13)	–	White matter structure	New classifier, using fractional anisotropy, mean diffusivity, and average fiber length, achieves an accuracy of 76%.
Lewis et al., 2014	24 months	113 (75/38)	31 (24/7)	23 (12/11)	–	White matter structure	(1) Local and global efficiency reduced in temporal, parietal, and occipital lobe in HR-ASD group; (2) Global efficiency only reduced in Broca’s area in HR-ASD group; (3) Inverse relationship between efficiency in all areas above and symptom severity in HR group.
Lewis et al., 2017	6, 12, and 24 months	184	31 (29/2)	76	–	White matter structure	(1) HR-ASD group was found to have network inefficiencies in the auditory-associated regions at 6 months and in regions associated with low-level processing and Broca’s area at 12 months; (2) In the HR group, inefficiencies in the primary and secondary auditory areas at 6 months and in the sensory integration areas at 12 months were associated with symptom severity at 24 months.
Elison et al., 2013	7 months	56 (31/15)	16 (11/5)	41 (24/17)	–	White matter structure	(1) Longer visual orienting latencies in HR-ASD; (2) Visual orienting latencies were associated with microstructure of the splenium of the corpus callosum in LR group but not HR or HR-ASD groups.
Liu et al., 2019	6 weeks and 18 and 36 months	19 (10/9)	–	15 (11/4)	–	White matter structure	(1) LR infants had higher FA in the left superior longitudinal fasciculus (SLF); HR infants had higher FA in the right SLF; (2) In both LR and HR infants, FA and lateralization in the SLF at 6 weeks was associated with language development at 18 months and ASD severity at 36 months.
Cárdenas-de-la-Parra et al., 2020	6, 12, and 24 months	341	56	162	–	White and gray matter structures	(1) While overall HR-ASD exhibit more brain growth than HR-TD and LR, they also exhibit increased WM growth and decreased GM growth relatively; (2) Distinction begins at around 12 months; (3) Increased growth in splenium of corpus callosum; (4) Decreased growth in right fusiform gyrus and right inferior temporal gyrus.
Darki et al., 2021	5 months	29 (17/12)	–	17 (6/11)	–	White and gray matter structure	T1w/T2w ratio reduced in HR infants compared to LR infants.
Shen et al., 2013	6, 9, 12, 15, 18, and 24 months	33 (22/11)	10	22 (15/7)	0	CSF and total brain volume	(1) The increased extra-axial fluid detected as early as 6 months of age was predictive of later ASD symptoms; (2) ASD group had a significantly faster growth trajectory of total cerebrum; (3) ASD group had significantly greater extra-axial fluid than all other groups.
Shen et al., 2017	6, 12, and 24 months	221 (137/84)	47 (42/5)	122 (76/46)	–	CSF and total brain volume	(1) HR-ASD infants had significantly greater extra-axial CSF volume at 6 months than HR-TD and LR infants; extra-axial CSF remained elevated through 24 months; (2) Infants with more severe autism symptoms had an even greater volume of extra-axial CSF from 6–24 months; (3) Extra-axial CSF volume at 6 months predicted ASD infants at 24 months with an overall accuracy of 69% and corresponding 66% sensitivity and 68% specificity.
Hazlett et al., 2017	6, 12, and 24 months	106	15	42	–	Brain volume	(1) HR-ASD infants exhibit greater cortical surface area expansion between 6–12 months; (2) HR-ASD infants exhibit brain volume overgrowth between 12-24 months; (3) A deep learning algorithm trained with cortical surface area information from HR infants predicted ASD diagnosis at 24 months (94% accuracy, 88% sensitivity, 95% specificity, 81% positive predictive value, 97% negative predictive value.
Hazlett et al., 2011	24 months and 4–5 years	–	59 (51/8)	38 (27/11)	–	Large regional volumes	(1) No differences in cortical thickness were observed in ASD toddlers; (2) A disproportionate enlargement in temporal lobe white matter in ASD toddlers.
Pote et al., 2019	4–6 and 36 months	24 (11/13)	4	26 (12/14)	–	Large regional volumes	(1) 4–6-month old infants at high-risk of ASD have larger cerebellum and subcortical volumes than low-risk infants. (2) Larger volumes in high-risk infants are associated with core repetitive behaviors in childhood
Hazlett et al., 2012	6 months	98 (61/37)	–	36 (21/15)	–	Cerebrum, cerebellum, ventricles	No group differences were observed for intracranial, cerebrum, cerebellum, or lateral ventricle volume or for head circumference.
Li et al., 2019	6, 12, and 24 months	61 (46/15)	30 (25/5)	215 (133/82)	–	Amygdala, hippocampal volume	The overgrowth of the amygdala and cornu ammonis sectors (CA) 1–3 start from 6 months of age, which may be related to the emergence of ASD.
Swanson et al., 2017	6, 12, and 24 months	382	86	143	–	Subcortical brain volume	Only HR children with early language delay or who later were diagnosed with ASD were found to have an association between their 12 months subcortical structures, including the caudate nucleus and amygdala, and language skills at 24 months.
MacDuffie et al., 2020	6, 12, and 24 months	305 (192/113)	71 (59/12)	127 (73/54)	–	Subcortical brain volume	(1) Problems with sleep onset were more common at the age of 6–12 months among infants who later developed ASD; (2) Sleep onset problems related to hippocampal volume trajectories from 6 to 24 months of only for high risk infants who developed ASD; (3) Brain-sleep relations were specific to hippocampus; no other significant relationships found with volume trajectories with other subcortical structures.
McKinnon et al., 2019	12 and/or 24 months	12 months: 87 24 months: 66	11 11	31 20	–	Resting state, global functional connectivity	Ritualistic and stereotyped behaviors were associated with differences in functional connectivity among the visual, default mode, control, and dorsal attention networks.
Emerson et al., 2017	6 and 24 months	59 (41/18)	11 (11/0)	0	0	Resting state, global functional connectivity	(1) Functional connectivity at 6 months correlated with social behavior, language, motor development, and repetitive behavior at 24 months; (2) A machine learning algorithm trained on functional connectivity at 6 months correctly predicted 9 of 11 ASD cases at 24 months.
Liu et al., 2020a	1.5 and 9 months	1.5 months: 33 (19/14) 9 months: 38 (23/15)	–	1.5 months: 32 (19/13) 9 months: 22 (11/11)	–	Functional connectivity	(1) Deficits in connectivity of networks, temporal and somatosensory, are related to integration of sensory and motor function in HR group at 1.5 months; (2) At 9 months, hyperconnectivity between auditory and somatosensory regions in HR group. (3) At 9 months, greater intrahemispheric connectivity between auditory cortex, temporal lobe, and hippocampus in LR group; (4) LR group presented with relatively large change toward increased long-range connectivity and reduced short-range connectivity compared to HR group.
Nair et al., 2021	6 weeks, 9 months, and 36 months	24 (16/12)	–	28 (13/11)	–	Structural and functional connectivity	(1) Thalamic-prefrontal underconnectivity in HR infants; (2) Thalamic-motor and thalamic-occipital overconnectivity in HR infants; (3) Aberrant connectivity predicted atypical social behavior at 9 and 36 months.
Blasi et al., 2015	4–7 months	15 (10/5)	–	18 (7/11)	–	Functional; auditory stimuli	(1) LR infants showed specialization for human voice processing in right temporal and medial frontal regions; (2) LR infants had stronger sensitivity to sad vocalizations in right fusiform gyrus and left hippocampus; (3) In HR group only, an association between social engagement and degree of voice specialization was found.
Liu et al., 2020b	9 and 36 months	27 (19/8)	–	16 (7/9)	–	Functional; auditory stimuli	(1) LR infants exhibited greater activity in left amygdala and left temporal regions, while listening to speech streams while sleeping; (2) LR infants had greater increase in functional connectivity between bilateral primary cortex and right interior insula; (3) Learning related signaling in the left temporal region was positively correlated with expressive language at 36 months; (4) In HR group, greater learning related signal increase correlated with less severe ASD symptomology at 36 months.

### Electroencephalogram Studies

An electroencephalogram (EEG) is an inexpensive, commonly used, typically non-invasive neurophysiological technique that monitors electrical activity in the brain by measuring the electric potential between electrodes that are placed on the scalp (Bell and Cuevas, 2012). The main application of an EEG is to assess cerebral function, as opposed to detecting structural abnormalities as in MRI. A total of 41 EEG studies met the inclusion criteria of this review. These articles have been organized into several sections based on analysis methods and main findings.

#### Event-Related Potential

##### Facial Processing

It has long been thought that differential activation patterns related to face processing and social bonding may be potential early signs of ASD. One potential difference in face processing in HR-ASD infants is hemispherical asymmetry. HR infants, and even more so in HR-ASD infants, exhibited aberrant left lateralization of event-related gamma-band phase coherence compared to LR infants when presented with familiar and unfamiliar faces (Keehn et al., 2015).

Electroencephalogram P400 is hypothesized to be related to face familiarization, but it’s potential relation to divergence in HR infant processing remains highly debated. In response to a novel face as a compared to a familiar face, both shorter P400 latency (Key and Stone, 2012a; Jones et al., 2016) in HR infants and no difference in P400 latency (Luyster et al., 2011, 2014; Guy et al., 2018) have been reported. To better understand the potential role of P400 in HR development, numerous stimuli have been tested. Elsabbagh et al. (2012) found that 7-month old infants who later developed ASD did not exhibit a difference in P400 amplitude in response to a change in gaze toward the infant as opposed to a gaze change away from the infant; both LR infants and HR who did not later develop ASD (HR-TD) exhibited significantly larger P400 amplitude in response to gazes toward the infant. In a follow-up study, Elsabbagh et al. (2015) found that greater P400 latency positively correlated with positive affect in infants interacting with mothers only in the HR group, indicating a more complex role of P400 in social bonding. P400 latency was found to be delayed in HR infants in response to direct gaze, and not in response to averted gaze (Elsabbagh et al., 2009, 2015). A recent study was able to leverage ERP data from 6-month-old infants undergoing a direct/averted gaze paradigm to classify HR-ASD and HR-TD with an accuracy of 88.44% (Abou-Abbas et al., 2021). ERP activation to different facial expressions has also been investigated (Key et al., 2015). Differences between LR and HR groups were found to be most pronounced for subtle face expressions. The HR group exhibited shorter P400 latency in response to small smiles compared to the LR group, which indicates that LR infants may process these facial expressions more substantially (Key et al., 2015). Interestingly, another study found that HR infants had faster responses to objects than the LR infants but equal responses to faces according to P400 latency, indicating that HR infants may process objects differently (McCleery et al., 2009). P400 amplitude and latency differences between HR and LR infants may be an early sign of ASD diagnosis that indicates divergence in facial processing.

N290, the EEG component precursor to the face-processing N170 component in adults, has also been studied in HR infants. Similar to P400, HR infants displayed faster N290 responses to objects than LR infants did (McCleery et al., 2009). This study also reported that HR infants lacked the hemispheric asymmetry for both P400 and N290 amplitude that LR infants exhibited in response to faces and objects. One study found that changes in the facial features of the visual stimulus were associated with the N290 component only in 9-month-old LR infants and not HR infants (Key and Stone, 2012b). In response to non-face stimuli, HR infants at 7 months who exhibited decreased N290 amplitude also had more atypical activity patterns and more severe autism symptoms (Shephard et al., 2020). Based on these two studies, the N290 component may be more important for facial processing in LR infants and may be more related to other types processing in HR infants.

Differences in P400 and N290 latency and amplitude between LR and HR infants indicate that HR infants are less sensitive to novel faces and subtle facial expressions, although there is not complete agreement in the field. An alternative hypothesis that HR infants have atypical processing of objects may be related to or partially explain atypical facial processing. Multiple studies have observed differences in other EEG components, including Nc and P100, but the relation between these components and facial processing is not well understood.

##### Auditory Processing

Event-Related Potential (ERP) evoked by auditory stimuli has also been investigated for its utility in early screening. One article found that ERP responses to non-native speech were similar between groups, but, between 6 and 12 months, LR infants exhibited smaller and more lateralized negative-going later slow wave (LSW) to the speech sounds than HR infants (Seery et al., 2013). Interestingly, another study found that at 12 months, HR-ASD showed reversed lateralization of LSW to speech stimuli compared to the LR group (Finch et al., 2017). A recent study found that HR individuals at 14 months, but not 10 months, showed attenuated frontal positive going activity across both hemispheres in response to their own name compared to an unfamiliar name (Arslan et al., 2020). HR infants may have atypical lateralization for auditory processing prior to 1-year-old.

Atypical auditory processing in HR infants has been linked to later development of ASD symptoms. In response to non-speech stimuli, HR-ASD infants exhibited mismatch response (MMR) latency compared to the LR group, and HR-ASD infants had overall larger P3 amplitude at 12 months (Riva et al., 2018). Both MMR latency and larger P3 amplitude were correlated with later developed ASD symptomology (Riva et al., 2018). In response to concatenated syllables, alpha coherence at 3 months was found to be associated with word production at 18-month-olds (Tran et al., 2021). In response to repeated tones, cortical gamma amplitude and inter-trial coherence in HR children were associated with reduced growth in language skills between 8 months and 3 years and elevated levels of parent-rated social communication symptoms at 3 years (Kolesnik et al., 2019). Atypical neural response to both speech and non-speech stimuli are associated with ASD symptoms, particularly language and social development, in ASD infants.

##### Sensory Processing

Children with ASD often have increased sensory hypersensitivity compared to typically developing children. A recent study found that the HR group showed greater sensory hypersensitivity at 6 months and 2 years, which was predictive of improved neural responsiveness and social outcomes at 4 years of age (Jones et al., 2018). Another study found that HR toddlers at 18 months had increased sensory seeking, generally indicating under-sensitivity, which was associated with atypical frontal asymmetry and social communication deficits (Damiano-Goodwin et al., 2018). Piccardi et al. (2021) reported that reduced neural repetition in response to tactile stimuli, rather than just under-sensitivity, predicted later ASD diagnosis (Piccardi et al., 2021). HR infants and toddlers exhibit diversely atypical sensory sensitivity, but hypersensitivity, in particular, may be associated with better social symptoms.

##### Form and Motion Processing

Differential neural activity during shape recognition and motion observation in HR infants may also be useful in early screening. One study found that HR infants showed increased lateral frontal positivity (LFP), an index related to high level processing and learning, and N700, an occipital component, response to low probability conditions of shape pairs, while LR infants demonstrated increased LFP and N700 response to the high probability shape pairs (Marin et al., 2020). Notably, LFP at 3 months across both groups predicted visual reception skills but not ASD symptoms at 18 months. Five-month-old HR infants, observing coherent visual motion, were found to have divergent lateralization of ERP activation patterns in response to global visual changes but minimal divergence in response to local visual changes (Nyström et al., 2020). The authors hypothesized that the findings support that differences in global coherence processing, rather than local processing, are responsible for divergent cognition in ASD.

#### Resting State Electroencephalogram

##### Functional Connectivity

The relationship between restricted and repetitive behaviors and functional connectivity was supported by two studies, a primary article and a replication of that study (Orekhova et al., 2014; Haartsen et al., 2019). In the original study, functional connectivity at 14 months had a strong correlation with the severity of restricted and repetitive behaviors at 3 years in response to audio-visual stimuli. HR-ASD infants also exhibited significantly elevated phase-lagged alpha-ranged connectivity compared to both LR infants and HR-TD infants (Orekhova et al., 2014). While the replication study failed to find significantly elevated global connectivity in HR-ASD infants, strong correlation between functional connectivity and restricted and repetitive was further supported (Haartsen et al., 2019). Conversely, another study found that 12-month-old HR-ASD infants showed reduced functional connectivity relative to LR and HR infants in response to language-relevant stimuli (Righi et al., 2014). A recent study has shed light on perhaps a more complicated explanation for these conflicting results. Dickinson et al. (2020) found that HR-ASD infants with lower frontal connectivity and higher right temporoparietal connectivity at 3 months predicted more severe ASD symptoms at 18 months (Dickinson et al., 2020).

##### Frequency Bands and Autism Spectrum Disorder Symptomology

Several studies have demonstrated correlations between frequency bands and ASD-related symptoms. Researchers found that frontal theta change in HR infants correlated with greater non-verbal skills and verbal skills in toddlerhood (Jones et al., 2020). For HR-ASD infants, theta EEG explained over 80% of variance in non-verbal skills at 3 years of age. Two studies found that expressive language in HR infants correlated with frontal power in the gamma and alpha bands relative to LR infants (Levin et al., 2017; Wilkinson et al., 2019). Among HR infants, increased frontal gamma was only marginally associated with ASD diagnosis but significantly associated with reduced expressive language ability (Wilkinson et al., 2019); reduced frontal high-alpha power at 3 months was also robustly associated with poorer expressive language at 12 months (Levin et al., 2017). Another study found that peak alpha frequency at 12 months, while not a good predictor of ASD risk, did correlate with non-verbal cognitive ability at 36 month-olds (Carter Leno et al., 2021). Page et al. (2020) examined EEG during NREM sleep. Researchers, despite finding topographically distinct decreases in fast theta oscillations and fast sigma (15–16 Hz) and an increase in beta oscillations in HR-ASD infants compared to LR infants, found no significant correlations between ASD severity and these frequency bands (Page et al., 2020).

Several bands in the frontal area may be correlated with a critical developmental skills, even if they cannot be directly linked to ASD diagnosis. For example, the theta band may be related to non-verbal skills, and the gamma and alpha bands to expressive language. On the other hand, frontal alpha asymmetry may be positively associated with several ASD-related behaviors.

#### Developmental Trajectory

Recent research has evaluated how early differences in HR neural activity may relate to children’s neural development over time. During resting state, HR infants exhibited frontal alpha asymmetry differently from LR infants at 6 months and opposite growth in asymmetry by 12 months (Gabard-Durnam et al., 2015). Similarly, found that at 12 months the developmental trajectories of HR-ASD and LR infants diverged (Bosl et al., 2011). A longitudinal study in HR and LR children from 3 to 36 months found that power dynamics during the first year and delta and gamma bands in particular were most robust in differentiating ASD diagnoses (Gabard-Durnam et al., 2019). Tierney et al. (2012) investigated the spectral power across delta, theta, low alpha, high alpha, beta, and gamma frequencies in infants/toddlers at 6, 9, 12, 18, and 24 months of age. Reduced spectral power across all frequency bands were observed in HR infants at 6 months, but only the delta, theta, and beta frequency bands were rectified by 24 months, indicating divergent trajectories for individual frequency bands in HR individuals (Tierney et al., 2012). Huberty et al. (2021) also found that HR infants had divergent trajectories for power in each band but found that reduced power at 3 months and steeper change during later development was associated with familial risk and not later ASD diagnosis (Huberty et al., 2021).

Increasingly, developmental trajectory of certain EEG components are being used to predict ASD diagnosis and severity. Wilkinson et al. (2020) used spectral power across six frequency bands over a period from 3 to 12 months of age to accurately predict language scores at 24 months (Wilkinson et al., 2020). Another study found that multiscale entropy, a measure of functional brain complexity, also exhibited a divergent developmental trajectory in HR children (Bosl et al., 2011). When using multiscale entropy as a classifier for ASD, accuracy peaked at 9 months for boys (100% accuracy) and 6 months for girls (80% accuracy) and decreased at later developmental timepoints. Recently, statistical learning methods were implemented to classify individuals developing ASD based on EEG analyzes at 3, 6, 9, 12, 18, 24, and 36 months (Bosl et al., 2018). Using parameters including frequency bands and multiscale entropy, this algorithm accurately classified HR-ASD infants at greater than 95% for certain ages and predicted ADOS severity scores that strongly correlated to future ADOS scores.

In summary, HR infants have distinct P400 and N290 activity in response to both face and object stimuli, although the exact differences and the interpretation of those differences is debated. Across many different paradigms, HR infants exhibit atypical lateralization of neural activity. Neural response to auditory stimuli and sensory stimuli in HR infants was found to predict language and social communication development. Global functional connectivity was found to be strongly correlated with repetitive and restricted behaviors. Developmental trajectories across different frequency bands in HR infants were found to predict different ASD-related symptoms and could be leveraged to classify later ASD diagnosis with high accuracy. See Table 2 for details about each EEG article reviewed.

**TABLE 2:** Electroencephalogram (EEG) studies on early screening of autism spectrum disorders.

Authors, year	Age	High-risk group		Control group		Stimuli/task	Main findings
		No. (M/F)	HR- ASD no.	No. (M/F)	LR-ASD no.		
McCleery et al., 2009	10 months	20 (12/8)	–	20 (11/9)	–	Facial processing	(1) HR infants responded faster to objects than LR infants (N290 and P400); (2) In HR, faster responses to objects than faces (N290 and P400); (3) Lack of hemispheric asymmetry in HR infants for P100, P400, and N290 amplitude
Luyster et al., 2011	12 months	32 (18/14)	–	24 (11/13)	–	Facial processing	(1) HR and LR showed similar responses in face-sensitive components; (2) A trend toward a larger P400 peak amplitude in the HR group than LR, but not the mean amplitude measurement.
Luyster et al., 2014	6, 9, 12, 18, 24, and 36 months	61	9	70	0	Facial processing	For both HR and LR infants: (1) Between 6 and 36 months of age, the amplitude of the P1 increases, the amplitude of the N290 decreases; (2) the P400 and Nc both show amplitude increases until 24 months, followed by decreases between 24 and 36 months; (3) P400 and Nc were both maximal over the right hemisphere.
Keehn et al., 2015	6 and/or 12 months	49	11	46	0	Facial processing	(1) HR infants showed an aberrant pattern of leftward lateralization of intra-hemispheric coherence [gamma-band (30–50 Hz) phase coherence] by 12 months; (2) HR-ASD infants had the greatest leftward asymmetry at 12 months.
Guy et al., 2018	12 months	21	–	21	–	Facial processing	According to the Nc component, LR infants displayed greater activation in response to unfamiliar faces and toys than to their mother’s face and own toy, whereas HR-ASD did not differentiate based on familiarity.
Elsabbagh et al., 2009	10 months	19 (10/9)	–	17 (10/7)	–	Facial processing	(1) HR group showed prolonged latency of occipital P400 in response to direct gaze; (2) In gamma band, HR showed late and less persistent on right temporal region.
Elsabbagh et al., 2012	10 months	54 (21/33)	17	50 (21/29)	–	Facial processing	The LR and HR-TD infants exhibited significantly larger P400 amplitude in response to gaze toward compared to gaze away stimuli. HR-ASD did not.
Elsabbagh et al., 2015	7 months	45 (20 /25)	–	47 (18 /29)	–	Facial processing	In both groups, infants with more positive affect exhibited stronger differentiation to gaze stimuli. This association was observed with the earlier P100 for LR but with the later P400 for HR.
Jones et al., 2016	6–24 months	43 (28/15)	–	45 (26/19)	–	Facial processing	(1) At 24 months, HR infants showed shorter epochs of visual attention, faster, however, less prolonged neural activation to faces; (2) At 6 months, HR infants showed delayed sensitization responses; (3) These differences were less apparent at 12 months.
Key and Stone, 2012a	9 months	15 (10/5)	–	20 (13/7)	–	Facial processing	(1) P400 response latency to strangers faces was only observed in LR group; (2) Shorter Nc response latency to the mother’s face was associated with better interpersonal skills across both groups.
Key and Stone, 2012b	9 months	15 (10/5)	–	20 (13/7)	–	Facial processing	Shorter N290 latency in response to changes in facial expression only in the LR group.
Key et al., 2015	9 and 15 months	16 (9/7)	–	15 (10/5)	–	Facial processing	For facial expressions, LR infants exhibited longer processing and greater attention resource allocation.
Shephard et al., 2020	7 months and 7 years	42 (15/27)	–	35 (14/21)	–	Facial processing	Atypical neural correlates of object processing, not face processing, were associated with abnormally decreased right lateralization of N170 amplitudes and social deficits in later childhood.
Abou-Abbas et al., 2021	6–10, 24, and 36 months	50 (20/30)	17 (11/6)	44 (15/29)	–	Facial processing	Using ERP data in response to direct and averted gaze, researchers were able to implement a support vector machine that accurately classified HR-ASD and HR-TD at 88.44% accuracy
Kolesnik et al., 2019	8, 14, 24, and 36 months	116 (64/52)	17 (14/3)	27 (14/13)	–	Auditory processing	(1) HR-ASD < HR-TD for repetition suppression of 40–60 Hz evoked gamma for repeated tones; (2) HR-ASD > HR-TD for 10–20 Hz inter-trial coherence (ITC) for repeated tones; (3) Within HR, cortical gamma amplitude and ITC were associated with reduced growth in language skills between 8 months and 3 years and worsened social deficits at 3 years.
Seery et al., 2013	6, 9, and/or 12 months	62 (32/30)	14	46 (21/25)	–	Auditory processing	(1) ERP response to non-native speech contrast were similar between groups; (2) Between 6 and 12 months, LR displayed a lateralized response (LSW) to the sounds, while HR failed to display this pattern.
Finch et al., 2017	12 or 36 months	90 (48/42)	23 (16/7)	73 (35/38)	–	Auditory processing	(1) At 12 months, ASD showed reversed lateralization to speech stimuli compared to LR; (2) There was no association between lateralization to speech at 12 months and handedness at 36 months in the LR and HR; (3) ASD infants with lateralization patterns more similar to LR at 12 months were stronger right-handers at 36 months.
Riva et al., 2018	12 months	20	–	22	–	Auditory processing	(1) HR showed delayed mismatch response latency compared to LR; (2) HR showed overall larger P3 amplitude compared to LR.
Arslan et al., 2020	5 – 6 months	26 (17/9)	–	25 (15/10)	–	Auditory processing	(1) LR infants no enhanced response ERP to own name; (2) At 14 months HR showed frontal positive going activity to own name compared to unfamiliar name and relative to LR group.
Tran et al., 2021	3 and 18 months	36 (23/13)	12 (ASD-concern)	27 (17/10)	2 (ASD-concern)	Auditory processing	(1) ASD-Concern had reduced left fronto-central phase coherence in the theta and alpha bands, in response to hearing concatenated syllables; (2) Alpha coherence at 3 months correlated with word production at 18 months.
Jones et al., 2018	18–30 months 6–18 months	– 61(31/33)	59 17	34 63(33/30)	–	Sensory processing Developmental trajectory	(1) HR group > sensory hypersensitivity at 2 years of age; predictive of increased neural responsiveness to social stimuli and social approach at 4 years; (2) Perceptual sensitivity in HR and LR infants at 6 and 12 months was associated with larger P1 amplitude to faces at 18 months.
Damiano-Goodwin et al., 2018	18 months	20 (8/12)	–	20 (10/10)	–	Sensory processing Developmental trajectory	(1) HR > LR for sensory seeking; (2) Within HR, increased sensory seeking was concurrently associated with reduced social orienting across groups and resting frontal asymmetry (alpha, beta, and gamma).
Piccardi et al., 2021	10 and 24 months	44	–	18	–	Sensory processing	In response to vibrotactile stimulation of the feet, reduced neural repetition at 10 months predicted ASD at 24 months.
Marin et al., 2020	3 and 18 months	19 (13/6)	–	21 (11/10)	–	Visual form processing	(1) Familial risk infants showed increased lateral frontal positivity (LFP) and N700 response to the probabilistic condition; (2) LR infants demonstrated increased LFP and N700 response to the deterministic condition; (3) LFP at 3 months predicted 18 months visual reception skills and not ASD symptoms.
Nyström et al., 2020	5 months	50 (26/24)	–	23 (9/14)	–	Motion processing	(1) Different topographical organization for global form and motion processing for HR infants compared to LR; (2) More lateral activation in HR group; (3) Activation patterns for local visual change were similar between groups.
Orekhova et al., 2014	12–17 months	28 (10/18)	10 (7/3)	26 (12/14)	0	Functional connectivity	(1) HR-ASD > HR-TD&LR for phase-lagged alpha-range connectivity; (2) Hyper-connectivity was most prominent over frontal and central areas; (3) Hyper-connectivity in HR-ASD infants at 14 months correlated with restricted and behaviors at 3 years.
Haartsen et al., 2019 (Replication study of Orekhova et al., 2014)	13–18 and 36 months	81 (47/34)	13 (11/2)	20 (11/9)	–	Functional connectivity	(1) Did not replicate the finding that global EEG connectivity associated with ASD diagnosis; (2) Did replicate the association between higher functional connectivity at 14 months and greater severity of restricted and repetitive behaviors at 36 months in HR-ASD infants.
Righi et al., 2014	6 and 12 months	28	5	26	0	Functional connectivity	(1) HR 12 months old ASD infants showed reduced functional connectivity relative to LR and HR infants who were not diagnosed with ASD; (2) Differences in functional connectivity found in LR and HR infants who did not develop ASD.
Dickinson et al., 2020	3 months	36 (23/13)	11	29 (18/11)	3	Functional connectivity	Lower frontal and higher right temporoparietal connectivity at 3 months was associated only with ASD symptoms at 18 months, not cognitive ability generally.
Levin et al., 2017	3 months	25 (14/11)	7	14 (9/5)	0	Resting state	(1) HR < LR for frontal power; (2) Reduced frontal high-alpha power at 3 months was robustly associated with poorer expressive language at 12 months.
Wilkinson et al., 2019	24 months	58 (31/27)	16 (11/5)	43 (24/19)	0	Resting state	(1) HR-TD < LR on baseline frontal gamma power (30–50 Hz); (2) Among HR, increased frontal gamma was only marginally associated with ASD diagnosis, but significantly associated with reduced expressive language ability (MSEL); (3) In LR, no association between gamma power and language was present.
Jones et al., 2020	12 months and 2, 3, and 7 years	14 18	5 7	106 (55/51) 16	– 0	Resting state	(1) Frontal theta change in 14 HR infants correlated with greater non-verbal skills at 24 months and verbal skills (controlling for 12 months on verbal skills); (2) Predictive relation to verbal and non-verbal skills measured at 2, 3, and 7 years were also found; (3) Infant theta EEG explained over 80% of variance in non-verbal skills at age.
Carter Leno et al., 2021	12–36 months	97	32	95	–	Resting state	While peak alpha frequency was found to be correlated with non-verbal cognitive ability, it was found to not be associated with autism risk.
Gabard-Durnam et al., 2019	3–36 months	102 (56/46)	31 (22/9)	69 (37/32)	–	Resting state Developmental trajectory	(1) Power dynamics during the first post-natal year best differentiate ASD diagnoses in the frontal EEG model. (2) Delta and gamma frequency power trajectories consistently distinguish infants with ASD diagnoses from others.
Page et al., 2020	13–30 months	–	7 (5/2)	13 (5/8)	–	NREM sleep	Topographically distinct decreased fast theta oscillations (5–7.5 Hz), decreased fast sigma (15–16 Hz), and increased beta oscillations (20–25 Hz) in ASD compared to TD.
Tierney et al., 2012	6, 9, 12, 18, and 24 months	65 6 months: 25 9 months: 30 12 months: 36 18 months: 24 24 months: 20	–	57 6 months: 34 9 months: 32 12 months: 23 18 months: 11 24 months: 10	–	Developmental trajectory	(1) 6 months HR < LR for spectral power across delta, theta, low alpha, high alpha, beta, and gamma frequency bands; (2) Different trajectories of change in spectral power in the subsequent developmental window.
Gabard-Durnam et al., 2015	6, 12, and 18 months	57 6 months: 25 (11/14) 12 months: 36 (18/18) 18 months: 24 (14/10)	–	51 6 months: 34 (15/19) 12 months: 23 (11/12) 18 months: 11 (5/6)	–	Developmental trajectory	(1) LR infants had more negative alpha asymmetry than HR infants; (2) HR and LR infants had opposite growth trajectories in asymmetry; (3) By 18 months, HR and LR alpha asymmetry was not significantly different.
Bosl et al., 2018	3, 6, 9, 12, 18, 24, and 36 months	99	32	89	3	Developmental trajectory	(1) A machine learning algorithm using 1026 EEG features gathered between 6–9 months classified HR-ASD infants with nearly 100% accuracy; (2) EEG features gathered between 3–9 months correlated strongly with autism symptom severity at 36 months; (3) HR-ASD and LR children have very different developmental trajectories, especially after the 12 month timepoint.
Wilkinson et al., 2020	3–24 months	72 (39/33)	21	58 (30/28)	–	Developmental trajectory	(1) EEG measures of power (theta and delta) were found to be correlated to language skills only in the HR group; (2) No significant language skill difference between the groups.
Bosl et al., 2011	6 – 24 months	46	–	33	–	Developmental trajectory	(1) Different developmental trajectory for multiscale entropy for HR relative to controls; (2) Differences greatest at 9–12 months.
Huberty et al., 2021	3–36 months	214	61	183	6	Developmental trajectory	(1) Familial risk, not later ASD diagnosis, was correlated with reduced power at 3 months and steeper change after that; (2) Difference in trajectory of spectral power was observed in all bands.

### Functional Near-Infrared Spectroscopy Studies

Functional near-infrared spectroscopy (fNIRS) is a non-invasive, low-cost, easy set-up functional neuroimaging technique that is used to measure frontal-temporal brain activity. It allows functional imaging of brain activation through the monitoring of blood oxygenation and volume in specific parts of the brain. This is done by measuring the concentration of both oxyhemoglobin and deoxyhemoglobin—which scatter near infrared light of different wavelengths (Di Domenico et al., 2019). In short, researchers are able to monitor blood flow in the brain by measuring the changes in near-infrared light. Compared with MRI and EEG, fNIRS is more tolerant to head movement; however, it is a relatively new tool and less commonly used. A total of eight published articles were selected that utilized near-infrared spectroscopy. Of the eight articles, three used simple language as the stimuli, while five used more complex social and non-social stimuli.

#### Language

In Keehn et al. (2013), Edwards et al. (2017) and Pecukonis et al. (2020), a trisyllabic sequence in either ABB or ABC was used as a stimulus (Keehn et al., 2013; Edwards et al., 2017; Pecukonis et al., 2020). HR-ASD infants at 3 months had increased functional connectivity but at 12 months had decreased functional connectivity compared to the LR infants (Keehn et al., 2013). While LR infants at 6 months had greater response activation in the temporal and frontal lobes compared to occipital and parietal lobes, HR infants did not (Pecukonis et al., 2020). LR infants also had greater activation in the frontal and parietal lobes than HR infants (Pecukonis et al., 2020). Female HR infants at 3 months had constant neural activity over exposure to repetition-based stimuli, rather than decreased activity as observed in female LR infants (Edwards et al., 2017).

#### Social Function

One study showed that HR infants between the ages of 4–6 months showed less selective neural responses to both auditory and visual social stimuli than LR infants (Lloyd-Fox et al., 2013). Other studies have shown that HR-ASD infants exhibited reduced brain activation when presented with a social task-based stimuli (Fox et al., 2013; Braukmann et al., 2018; Lloyd-Fox et al., 2018; Bhat et al., 2019). More specifically, HR-ASD infants showed reduced oxyhemoglobin concentration in the lateral regions and right posterior temporal cortex and increased deoxyhemoglobin in the frontal region relative to LR infants (Fox et al., 2013; Braukmann et al., 2018). Furthermore, reduced activation to visual social stimuli across the inferior frontal region and posterior temporal regions were shown in HR-ASD infants (Lloyd-Fox et al., 2018). Parallel to this, two studies that compared response to social and non-social interactions in their analyses found that HR-ASD infants showed greater brain activity when presented with non-social tasks (Lloyd-Fox et al., 2018; Bhat et al., 2019). In Lloyd-Fox et al. (2018), HR-ASD infants exhibited increased activation for non-social stimuli within the left lateralized temporal regions compared to the LR and HR infants. These researchers also reported that these differences in brain activity correlated significantly with parent-rated ASD symptoms at 3 years of age. In Bhat et al. (2019), functional connectivity was found to be enhanced in HR infants in pre- and post- social periods, but reduced during social periods compared to LR infants (Bhat et al., 2019).

In summary, these fNIRS studies connected brain activity and language and social communication development in HR infants. In response to trisyllabic stimulus, HR infants at 3 months had hyperconnectivity, at 6 months had decreased activation in the frontal and parietal lobes, and at 12 months had underconnectivity. HR infants exhibited greater activation in response to non-social tasks and decreased activation for visual social stimuli. See Table 3 for details about each article reviewed.

**TABLE 3:** Functional near-infrared spectroscopy (fNIRS) studies on early screening of autism spectrum disorders.

Authors, year	Age	High-risk group		Control group		Stimuli/task	Main findings
		No. (M/F)	HR - ASD no.	No. (M/F)	LR - ASD no.		
Keehn et al., 2013	3, 6, 9, and 12 months	27	–	37	–	Language	(1) 3-month HR > LR for overall functional connectivity; (2) 12-month HR < LR for connectivity.
Edwards et al., 2017	3 months	21 (13/8)	–	17 (10/7)	–	Language	Female LR showed initial neural activation that decreased over exposure to repetition-based stimuli, while female HR showed no changes in neural activity.
Pecukonis et al., 2020	6 and 24 months	14 (7/7)	5	18 (9/9)	–	Language	(1) While LR infants at 6 months had greater response activation in the temporal and frontal lobes compared to occipital and parietal lobes, HR infants did not; (2) LR infants, compared to HR-ASD infants at 6 months, had greater activation in the frontal and parietal lobes.
Fox et al., 2013	6–8 months	10 (4/6)	–	10 (4/6)	–	Social communication	(1) HR < LR for oxy-hemoglobin responses in lateral regions; (2) HR > LR for deoxy-hemoglobin responses in frontal channels; (3) The oxyhemoglobin response in the orbitofrontal cortex was only significantly different between seeing their mother smiling and a smiling stranger in the LR group
Lloyd-Fox et al., 2013	4–6 months	18 (8/10)	–	16 (10/6)	–	Social communication	(1) HR infants between 4 and 6 months showed less selective neural responses to auditory and visual stimuli relative to LRC; (2) Responses to the visual social stimuli revealed significant hemodynamic increase in HbO2 over the posterior STS region in both HR and LR groups; however, response in LRC was more extensive; (3) LR had greater hemodynamic response to vocal stimuli relative to non-vocal stimuli in right hemispheres (anterior STS region); (4) HR significant effect only for non-vocal condition relative to local condition.
Lloyd-Fox et al., 2018	4–6 months	20 (10/10)	5 (3/2)	16 (10/6)	–	Social communication	(1) HR-ASD decreased activation in response to visual social stimuli across inferior frontal and posterior temporal regions; (2) Reduced temporal left lateralization in HR-ASD in response to vocal stimuli. (3) HR-ASD > LR/HR-TD for activation to non-vocal sounds within left lateralized temporal regions.
Braukmann et al., 2018	5 months	16 (9/7)	–	13 (4/9)	–	Social communication	HR < LR for activation to social stimuli in the right posterior temporal cortex.
Bhat et al., 2019	6–9 months	9 (7/2)	–	6 (1/5)	–	Social communication	(1) Cortical hyper-connectivity in the first year precedes overt signs of ASD seen in the second year; (2) HR infants had reduced bilateral hemispheric functional activation in the social period; (3) HR infants had greater functional connectivity in the non-social period; (4) Early differences in functional activation and connectivity may be associated with ASD risk.

## Discussion

To this day, psychometric testing remains the main tool for the screening and diagnosis of ASD. Multiple review articles have been written about psychometric early screening tools (Towle and Patrick, 2016; Thabtah and Peebles, 2019; Levy et al., 2020). However, psychometric testing has inherent limitations, which precludes application in infants. Recently, biological testing and its value in early screening has been explored and reported (Frye et al., 2019). Among these biomarkers, differences in brain structure and function in ASD are being identified at earlier stages of development. Neuroradiological and neurophysiological testing via MRI, EEG, and fNIRS have increasing promise as screening tools in infancy with high specificity and sensitivity. This systematic review is the first to provide comprehensive discussion of these neuroimaging and electrophysiological findings in infants at risk for ASD.

The findings in the present systematic review demonstrate how different research tools can synergize and contribute to a common research question. MRI, a sophisticated but commonly used technique, can produce high resolution images with dense information about structure and function. The acquisition of high quality MRI images in any infant population challenging but is becoming more feasible (Denisova, 2019). Notably, the research teams that were able to scan hundreds of patients, such as Shen et al. (2017) and Wolff et al. (2017), were also studies with definitive findings, which gives hope that future research can produce more statistically and clinically meaningful results (Shen et al., 2017; Wolff et al., 2017). EEG and fNIRS are comparatively more affordable and easier to administer to infants. The convenience and temporal resolution of EEG and fNIRS suit them well to more complex experimental paradigms that involve stimuli and social interaction. As our understanding of how ASD brain processing and development may differ from typically developing people grows, EEG and fNIRS experimental design will evolve as well. We propose that the use in combination of these imaging and physiological methods and the use of deep learning algorithms will increase sensitivity and specificity of ASD development prediction.

In order to better understand the underlying mechanisms of ASD, it is important to identify biological differences that are clinically meaningful. Certain structural and functional differences have been implicated in the development of certain ASD-related behaviors. For example, subcortical brain volumes between 4 and 6 months (Pote et al., 2019), FA in the callosal pathways at 6 months (Wolff et al., 2015), and EEG functional connectivity at 14 months (Orekhova et al., 2014; Haartsen et al., 2019) were all associated with repetitive behaviors. Regarding language processing, MRI studies implicated network inefficiencies in auditory-associated regions (Liu et al., 2020a), and ERP studies implicated atypical lateralization and activity in response to diverse auditory stimuli (Seery et al., 2013); each of these findings were independently associated with language development. Future research that clarifies the relationship between the brain of children with ASD and the symptoms they experience will be invaluable in better understanding and screening for ASD at a young age.

Brain structure in HR infants at 6 months may alone be a reliable predictor for later ASD diagnosis. Several studies found at 6 months old that HR infants have divergent white matter tract development (Wolff et al., 2015), increased extra-axial fluid (Shen et al., 2013, 2017), greater volumes in the corpus callosum, amygdala, and hippocampus (Li et al., 2019), network inefficiencies in auditory-associated regions (Lewis et al., 2017), and cortical surface area hyper-expansion (Hazlett et al., 2017). There is even evidence that these structural differences are directly related to the ASD pathology; however, only a few, recent articles have investigated structure-symptom severity relationship. Nevertheless, preliminary, machine-learning-based prediction of ASD diagnosis using structural MRI data conducted by Hazlett et al. (2017) screened HR infants with high accuracy (81%). Future research into the structural differences in HR infants can improve ASD screening accuracy.

Several motifs in atypical brain function were identified in HR infants across functional imaging and neurophysiological testing techniques. First of all, HR-ASD infants have reduced specialization for social stimuli compared to LR infants. MRI research found that HR infants had diminished specialization for human voices in the temporal and medial regions (Blasi et al., 2015; Liu et al., 2020b). EEG research analyzing the P400 and N290 components has shown that HR infants express relatively shortened reaction to novel faces (Key and Stone, 2012a; Jones et al., 2016) and changes in facial expression (Key et al., 2015). Multiple fNIRS studies showed HR infants had reduced activity for social stimuli (Fox et al., 2013; Braukmann et al., 2018; Lloyd-Fox et al., 2018; Bhat et al., 2019). On a related note, EEG (McCleery et al., 2009; Shephard et al., 2020) and fNIRS evidence (Lloyd-Fox et al., 2018) supports that HR-ASD infants may also have atypical reaction to non-social stimuli. Second, HR-ASD infants exhibit atypical lateralization of brain function, especially in response to auditory stimuli. fNIRS research revealed that HR-ASD infants exhibited reduced lateralization of brain activity in response to visual social stimuli (Lloyd-Fox et al., 2018), and EEG research revealed different laterization in response to facial (McCleery et al., 2009), auditory (Seery et al., 2013; Finch et al., 2017), and sensory-somatic stimuli (Damiano-Goodwin et al., 2018). Finally, functional connectivity was found to be aberrant, based on findings from MRI (Liu et al., 2020a; Nair et al., 2021), EEG (Dickinson et al., 2020), and fNIRS studies (Bhat et al., 2019). There is some agreement that HR infants between 6 and 12 months have hyperconnectivity in temporoparietal pathways (Dickinson et al., 2020; Liu et al., 2020a), but functional connectivity was found to vary greatly depending on developmental timepoint, even in the same brain region.

Nevertheless, functional measures have been successful in predicting ASD diagnosis. Emerson et al. (2017) used fMRI-based functional connectivity to predict ASD with an accuracy of 96.6%. Bosl et al. (2011) and Wilkinson et al. (2020) were able to use multiple EEG parameters, such as spectral power across multiple frequency bands and multiscale entropy, in infants to predict ASD development with high accuracy. From this systematic review, we foresee great value in future directions that combine multiple approaches to optimize screening. Biological diagnostic technologies in combination with deep learning and artificial intelligence prediction models have potential for impactful clinical application in ASD detection during infancy. There are clear advantages compared with most psychometric screening methods in terms of detection time and accuracy for ASD, which warrant urgent attention and further research.

Ultimately, leveraging modern technologies for the early screening of ASD allows for highly accurate methods for classification even in infants. Early diagnosis allows for early initiation of therapeutic intervention that may exert beneficial effects for the rest of an individual’s life. It has been widely hypothesized that a developmental critical period exists for ASD, after which time intervention has reduced effectiveness (LeBlanc and Fagiolini, 2011); therefore, early intervention may prove to be especially important for ASD. Psychometric testing relies on the perception and interpretation of symptoms and will always be limited as a result. As described in this systematic review, neuroimaging and neurophysiological methods mainly MRI, EEG, and fNIRS can detect potential characteristics of ASD as early as 1.5 months after birth. Genetic, immunological or metabolic biomarkers may indicate pathogenetic features in ASD individuals and should be checked in combination with these neuroimaging and electrophysiological tests (Frye et al., 2019). It is imperative that empirical methods for early screening of ASD are rigorously pursued in the future, in order to improve the timing and reliability of ASD diagnosis.

In conclusion, certain neuroimaging and electrophysiological features in infancy can be effectively assayed to predict later ASD diagnosis. By elucidating these distinctions and directly correlating them with ASD core symptoms, we improve our understanding of ASD pathology and our ability to diagnose ASD at younger ages. MRI, EEG, and fNIRS each have great potential to improve the accuracy and timeline of ASD diagnosis by use in combination with conventional psychometric methods and other biologically-grounded tests, such as genetic testing. This study is limited as we did not conduct any quantitative assessment of the research database. Also, we focused on neuroimaging and neurophysiological features; we did not search or compare with other quantitative tests, such as genetic or immunological markers, eye tracking, or robotic technology. Further studies are warranted to validate and implement neuroimaging and electrophysiological techniques to meet the urgent needs for early diagnosis and early intervention.

## Data Availability Statement

The original contributions presented in the study are included in the article/supplementary material, further inquiries can be directed to the corresponding author.

## Author Contributions

X-JK: conceptualization and funding acquisition. CC, JW, ST, HS, and MZ: investigation. CC, JW, and ST: writing—original draft preparation. CC, JW, HS, and X-JK: writing—review and editing. CC, JW, and ST: tables and figures. All authors have read and agreed to the published version of the manuscript.

## Conflict of Interest

The authors declare that the research was conducted in the absence of any commercial or financial relationships that could be construed as a potential conflict of interest.

## Publisher’s Note

All claims expressed in this article are solely those of the authors and do not necessarily represent those of their affiliated organizations, or those of the publisher, the editors and the reviewers. Any product that may be evaluated in this article, or claim that may be made by its manufacturer, is not guaranteed or endorsed by the publisher.
